# Osteoblast Cell Response on the Ti6Al4V Alloy Heat-Treated

**DOI:** 10.3390/ma10040445

**Published:** 2017-04-23

**Authors:** Mercedes Paulina Chávez-Díaz, María Lorenza Escudero-Rincón, Elsa Miriam Arce-Estrada, Román Cabrera-Sierra

**Affiliations:** 1 Departamento de Ingeniería en Metalurgia y Materiales, Instituto Politécnico Nacional (ESIQIE-IPN), UPALM Zacatenco, Ciudad de México 07738, Mexico; mpaulinachavezdiaz@yahoo.com (M.P.C.-D.); earce@ipn.mx (E.M.A.-E.); 2 Departamento de Ingeniería de Superficies, Corrosión y Durabilidad, Centro Nacional de Investigaciones Metalúrgicas (CENIM-CSIC), Madrid 28040, Spain; escudero@cenim.csic.es; 3 Departamento de Ingeniería Química Industrial, Instituto Politécnico Nacional (ESIQIE-IPN), UPALM Zacatenco, Ciudad de México 07738, Mexico

**Keywords:** Ti6Al4V, biomaterials, microstructure, osteoblasts, heat treatment, titanium oxide

## Abstract

In an effort to examine the effect of the microstructural changes of the Ti6Al4V alloy, two heat treatments were carried out below (Ti6Al4V_800_) and above (Ti6Al4V_1050_) its β-phase transformation temperature. After each treatment, globular and lamellar microstructures were obtained. Saos-2 pre-osteoblast human osteosarcoma cells were seeded onto Ti6Al4V alloy disks and immersed in cell culture for 7 days. Electrochemical assays in situ were performed using OCP and EIS measurements. Impedance data show a passive behavior for the three Ti6Al4V alloys; additionally, enhanced impedance values were recorded for Ti6Al4V_800_ and Ti6Al4V_1050_ alloys. This passive behavior in culture medium is mostly due to the formation of TiO_2_ during their sterilization. Biocompatibility and cell adhesion were characterized using the SEM technique; Ti6Al4V as received and Ti6Al4V_800_ alloys exhibited polygonal and elongated morphology, whereas Ti6Al4V_1050_ alloy displayed a spherical morphology. Ti and O elements were identified by EDX analysis due to the TiO_2_ and signals of C, N and O, related to the formation of organic compounds from extracellular matrix. These results suggest that cell adhesion is more likely to occur on TiO_2_ formed in discrete α-phase regions (hcp) depending on its microstructure (grains).

## 1. Introduction

Commercially pure titanium (CP Ti) and titanium-based alloys are used in dental applications, joints, orthopedic trauma and reconstruction surgery and attachment systems due to their mechanical properties, resistance to corrosion and biocompatibility [1,2,3,4,5,6]. The corrosion resistance of Ti-based alloys is a result of a titanium oxide film formed on their surface at room temperature, which provides them with protection against biological fluids. However, due to its low thickness of between 1 and 4 nm, this film is very susceptible to fracture, leaving the metallic substrate exposed to body fluids and thus giving rise to base metal pitting and its later passivation [7,8,9,10]. Successive fracture-repassivation events of the layer lead to the release of metal ions and oxide particles that may affect important properties, such as Young’s modulus of the oxide and the substrate, the hardness and thickness of the oxide film, and its adherence [11]. Moreover, surface morphology and chemistry of the oxide film may be affected as well [12]. Electrochemical properties of the oxide film and its long-term stability in biofluids play an important role in biocompatibility of titanium and its alloys [13,14,15]. Titanium, aluminum and vanadium ions are released in the corrosion process, inhibiting the formation of apatite on the material, rising to a non-harmonious behavior between the implant and the bone [16,17,18].

In order to improve the corrosion resistance of these materials, which implies reduction in releases of toxic metal elements such as vanadium, present in Ti6Al4V alloy, as well as their biocompatibility, resistance to fatigue and appropriate Young’s modulus, they are subjected to a variety of treatments. These include mechanical, chemical, physical, thermal and heat treatments [19,20], thermomechanical [21] and deep cryogenic treatments [22], coatings [23,24,25], alkali-plus-heat [26], ion implantation [27,28,29], plasma spray [30], laser metal deposition (LMD) [31], selective laser melting and laser remelting (SLM) [32]. Heat treatments, in particular, may cause changes in the microstructure of the material depending on both temperature and cooling velocity, as well as on aging and alloy elements.

Ti6Al4V is an alpha-beta titanium alloy, where Al and V act as stabilizers of α and β phases, respectively, modifying the Ti transformation temperature; this temperature is 980 ± 20 °C [33,34] and the alloy may present two different microstructures: globular and lamellar, which provide mechanical and corrosion resistance to the Ti alloy [19,20,27,35,36,37]. Different structural morphologies may be transferred to or may have influence on the surface layers of the alloy, which could lead to different biological behaviors from those of unmodified materials. This study evaluated the effect of microstructural changes generated in Ti6Al4V alloy by two heat treatments, at 800 °C and 1050 °C—temperatures that are below and above the transformation temperature of Ti6Al4V alloy, respectively—on its biocorrosion behavior and its biocompatibility in the presence of osteoblastic cells in a culture medium.

## 2. Results and Discussion

### 2.1. Microstructural Characterization

**Figure 1:**
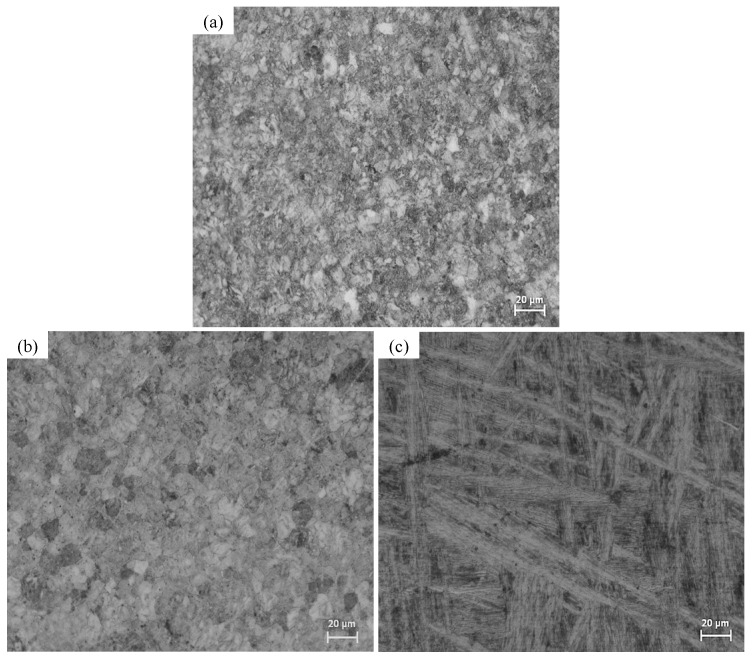
Micrographs of the following alloys: (**a**) Ti6Al4V as received; (**b**) Ti6Al4V_800_ treated at 800 °C; (**c**) Ti6Al4V_1050_ treated at 1050 °C.

Figure 1 shows the microstructural characterization of Ti6Al4V as received, and heat-treated at 800 °C (Ti6Al4V_800_) and 1050 °C (Ti6Al4V_1050_), respectively. The Ti6Al4V as received and Ti6Al4V_800_ alloys (Figure 1a,b) show β-phase globular grains (dark regions) sized between 2 and 4 μm in diameter, dispersed in the α-phase matrix (bright regions) of 5 to 8 μm in diameter. The α-phase acts as a barrier that prevents the grain size from increasing [33,34,35]. Meanwhile, for Ti6Al4V_1050_, Figure 1c shows a Widmanstatten type microstructure with acicular α-phase or fine α-phase plates surrounded by beta phase on grain edges [38,39]; the plate thickness is approximately 1 μm.

### 2.2. X-ray Diffraction Analysis (XRD)

**Figure 2:**
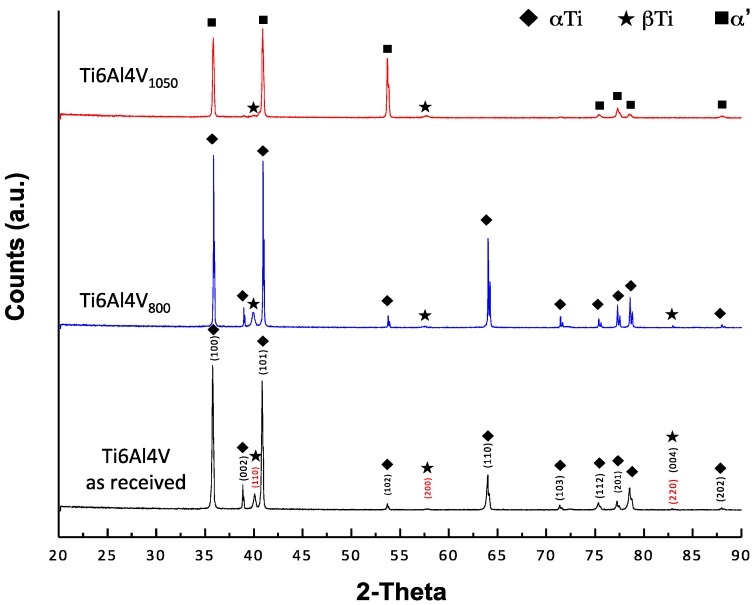
X-ray diffraction patterns (XRD) for Ti6Al4V alloys.

Figure 2 shows diffraction patterns obtained for the three alloys tested. All reflections of αTi and βTi can be observed for Ti6Al4V as received and Ti6Al4V_800_; whereas α’ (acicular α) is generated for Ti6Al4V_1050_ alloy [40]. Also, β-phase is observed to a smaller extent for the three materials, retained after the treatment at 38.88°. This phase remains stable in the alloy as a result of the redistribution of alloy elements (Al and V) during the cooling [41,42]. In general, the composition of the Ti6Al4V alloys after different heat treatments is mainly α-phase with a small amount of β-phase [43], with these alloys exhibiting different microstructural features (Figure 1); an acicular type for the Ti6Al4V_1050_ alloy, as opposed to grains for Ti6Al4V as received and Ti6Al4V_800_ alloys.

### 2.3. X-ray Photoelectron Spectroscopy Analysis (XPS)

**Figure 3:**
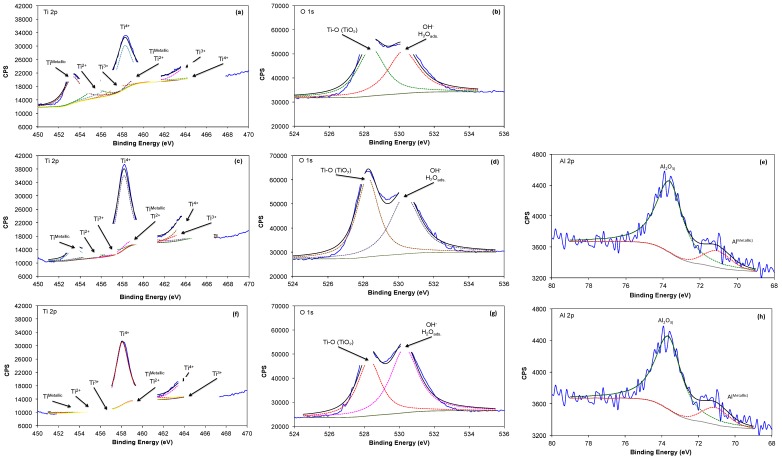
XPS spectra of Ti, O and Al measured at a surface for Ti6Al4V as received (**a**,**b**), Ti6Al4V_800_ (**c**–**e**) and (**f**–**h**) Ti6Al4V_1050_.

Figure 3 compares high-resolution XPS spectra of Ti 2p, O1s and Al 2p obtained on the surface of Ti6Al4V alloy, as received and with different heat treatments. The Ti 2p spectra (Figure 3a,c,f) can be fitted with four doublets and different binding energies. The first doublet, located at 453.7 and 460.3 eV is associated with the presence of Ti in the metallic state (Ti^Metallic^); the second, at 454.7 and 460.2 eV, may be assigned to the presence of TiO (Ti^2+^), and the third, at 457.4 and 464.2 eV, reveals the presence of Ti_2_O_3_ (Ti^3+^). The doublet with the highest intensity is observed at 458.4 and 463.6 eV, which could be attributed to the presence of TiO_2_ (Ti^4+^). These titanium oxides make part of a thin passive layer (a few nm thick) formed after the sterilization process at the outermost surface of the alloy.

The O 1s spectrum (Figure 3b,d,g) could be fitted with two components of similar intensity. The first one is located approximately at 529.6 eV and normally assigned to the presence of Ti-O bonds and related to TiO_2_. The second component in the O 1s spectrum is located at 531.5–532 eV and is attributed to the presence of OH^−^ groups, or to adsorbed water and a component with a binding energy of 531.8 eV, associated with the presence of oxygen in the form of aluminum oxide (Al_2_O_3_) [44]. This indicates that the oxide surface is mainly composed of TiO_2_ being hydrated and probably forming an oxy-hydroxide. The presence of aluminum in the chemical composition of the film was detected as Al^Metallic^ at 71 and 71.5 eV, and as Al_2_O_3_ at 74.2–74.8 eV, only for the Ti6Al4V_800_ and Ti6Al4V_1050_ alloys (Figure 3e,h) ; whereas for the Ti6Al4V as received, this signal is absent, likely due to its minor oxide thickness or contribution in the oxide. It is important to note that vanadium was not detected under the employed conditions [45,46,47,48]; however, it had been reported in low concentrations compared to oxygen.

The thickness of the oxide film on the metallic surfaces is calculated using the Strohmeier equation (Equation (1)) [49]:

*d*_o_ (nm) = *λ*_oxide_ sin(θ)ln{[(*I*_oxide_)(*λ*_metal_)(N_m_)]/[(*I*_metal_)(*λ*_oxide_)(N_o_)]+1} (1)

where *d_o_* is the thickness of the TiO_2_ layer (in nm); θ is the photoelectron output angle; *I*_metal_ and *I*_oxide_ are the intensities of the titanium components in the metallic state and as Ti^4+^ from the Ti2p peak; *λ*_metal_ and *λ*_oxide_ are the mean free paths of photoelectrons in the substrate and the oxide layer; and *N_m_* and *N_o_* are the volume densities of titanium atoms in metal and oxide. The values of *λ*_metal_ and *λ*_oxide_ are 1.73 and 3.08 nm, respectively [50]. Table 1 shows the oxide film thickness calculated by Equation (1), where Ti6Al4V_800_ and Ti6Al4V_1050_ exhibit an increase by a factor of 2 as compared to Ti6Al4V as received; this increase is observed for surfaces heat-treated at 800 °C and 1050 °C.

### 2.4. Electrochemical Characterization

#### 2.4.1. Evolution of the Open Circuit Potential (OCP)

**Figure 4:**
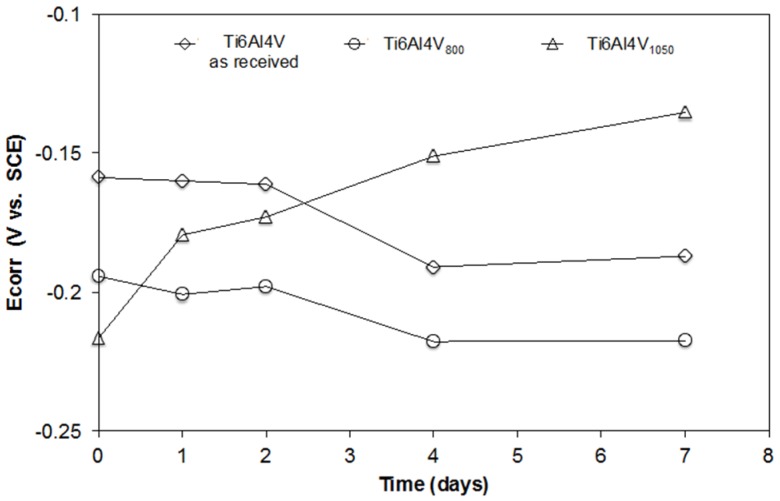
Corrosion potential (*Ecorr*) measurements of Ti6Al4V as received and heat-treated alloys over immersion time in the culture medium with osteoblasts cells.

Figure 4 shows the evolution of the open circuit potential (OCP) for Ti6Al4V as received, Ti6Al4V_800_ and Ti6Al4V_1050_ over the immersion time in a biological solution with osteoblastic cells (DMEM at 10% of FBS + cells). At *t* = 0 (culture medium without cells), the OCP values are seen to be more negative after the heat treatment of the alloy. The OCP values tend to displace in the negative direction and remain constant as of the 4th day for Ti6Al4V as received and Ti6Al4V_800_ alloys, showing similar activity to that of the passive oxide layer during its immersion. Conversely, the initial OCP of Ti6Al4V_1050_ alloy is more negative but its evolution during the test goes in a positive direction, improving over time without stabilizing at the end of the test (7 days). This trend shows that the surface layer formed on this alloy evolves towards a higher passivity due to the increase in the oxide thickness, a greater hydration and/or a positive interaction of proteins from the medium and likely a faster cell growth.

#### 2.4.2. Electrochemical Impedance Spectroscopy Characterization (EIS)

**Figure 5:**
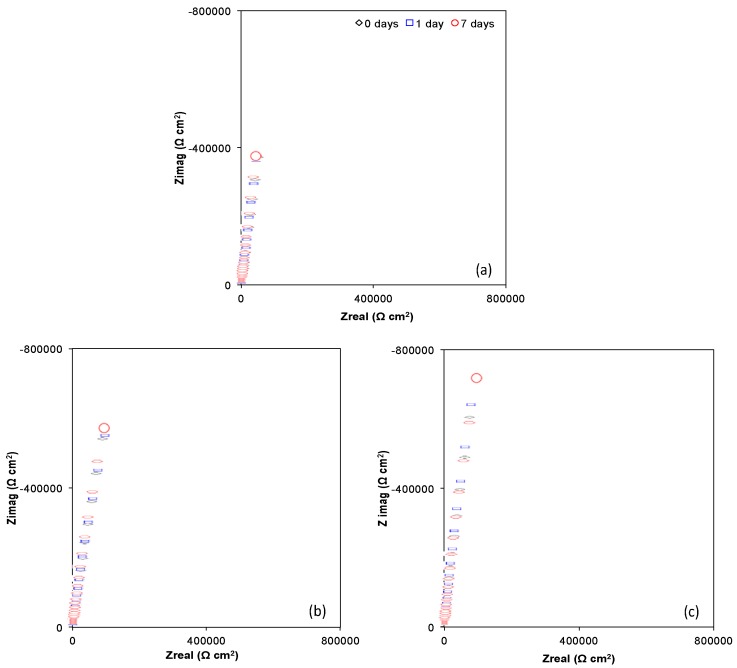
Nyquist plots (Zimag vs. Zreal) of the (**a**) Ti6Al4V as received; (**b**) Ti6Al4V_800_ and (**c**) Ti6Al4V_1050_ with the time in the culture medium with osteoblasts cells.

**Figure 6:**
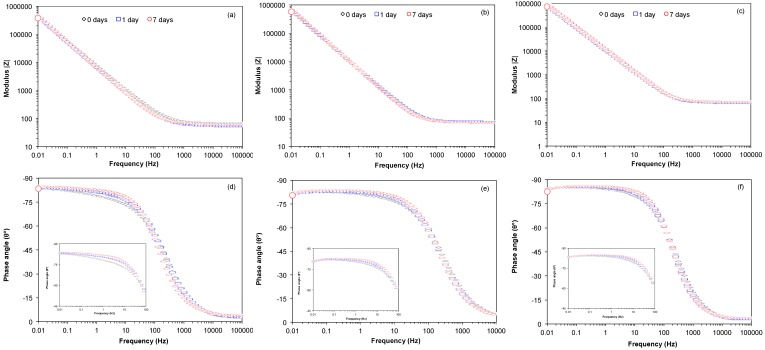
Bode plots (Modulus vs. Frequency and |Z| Phase angle vs. Frequency) recorded for Ti6Al4V as received (**a**,**d**), Ti6Al4V_800_ (**b**,**e**) and Ti6Al4V_1050_ (**c**,**f**) through the immersion time in the culture medium with osteoblasts cells.

Figure 5 and Figure 6 show Nyquist (Zimag vs. Zreal) and Bode plots (Module |Z| vs. Frequency and Phase angle vs. Frequency) obtained for Ti6Al4V as received, Ti6Al4V_800_ and Ti6Al4V_1050_ over the immersion time (0, 1 and 7 days) in the culture medium with osteoblastic cells (DMEM at 10% of FBS + osteoblastic cells). A similar electrochemical behavior can be observed for the different alloys during their immersion in the cell culture, which indicates a steady state over time due to the passivity provided by the oxide layer, mostly TiO_2_ (Figure 3). The capacitive response is due to an increase in imaginary impedance, Figure 5, related to the high corrosion resistance of the materials. This increase is higher for Ti6Al4V_800_ and Ti6Al4V_1050_ (Figure 5b,c), as compared to Ti6Al4V as received (Figure 5a), mainly due to the increase in the oxide thickness (see Table 1).

Bode plots of Impedance Modulus vs. Frequency (Figure 6a–c) reveal a plateau at high frequencies, associated with the resistance of the solution. The decrease in frequency gives rise to a slope between 0.887 and 0.936, which is attributed to a capacitive behavior; this is consistent with Phase angle vs. Frequency plots (Figure 6d–f), due to the increase in the angle from 0° to closely −90°. This capacitive response is associated with the presence of TiO_2_; furthermore, for 1 and 7 days of immersion a slight increase in phase angle values is observed (see inset) likely due to the adsorption of proteins and cells on the surface of the materials [51,52,53,54]. This latter may modify the relaxation of different time constants (possibly overlapped) in the interval from 10^−2^ to 10^3^ Hz for Ti6Al4V as received, Ti6Al4V_800_ and Ti6Al4V_1050_ alloys; allowing the identification of at least two time constants. According to the literature [55], these constants are associated with the formation of the film made of oxides, mainly TiO_2_, as well as of the proteins adsorbed on the oxide; the extracellular matrix and cells adhered to this latter.

**Figure 7:**
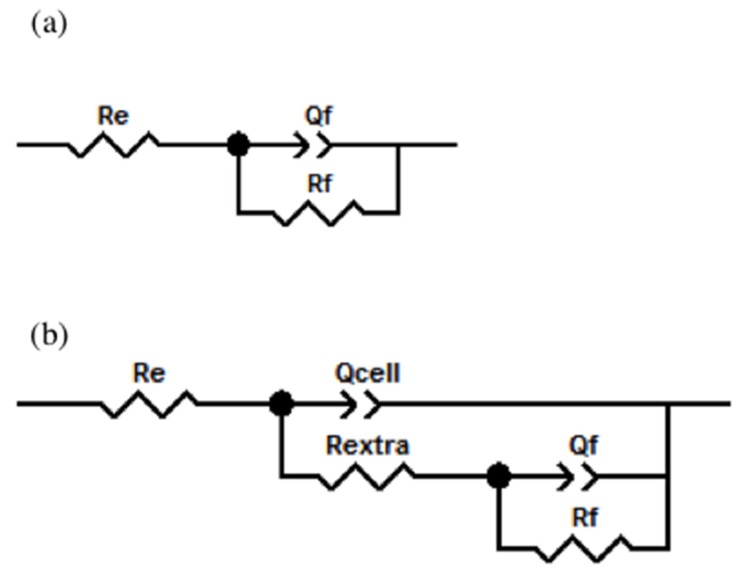
Equivalent circuits proposed for the fit of all impedance plots obtained for Ti6Al4V as received, Ti6Al4V_800_ and Ti6Al4V_1050_ alloys (**a**) without cells and (**b**) with osteoblastic cells.

Taking into consideration that the cell interaction until their confluence on the metallic surface (quasi-total surface coverage by cells) occurs locally, the impedance responses might be simulated considering a partially-covered surface. Impedance plots were fitted considering equivalent circuits shown in Figure 7. For the initial time given in Figure 7a, we have considered a circuit, RC, composed of electrolyte resistance *R_e_*; a constant phase element *Q_f_*, which simulates a non-linear behavior of the capacitor due to the passive film formed by the oxide and adsorbed proteins; and the resistance associated with this film, *R_f_*. Figure 7b exhibits the equivalent circuit used to simulate EIS plots for 1 and 7 days of immersion in osteoblast culture. In this case, the elements associated with the resistance and non-ideal capacitance of the extracellular matrix and osteoblasts, *R_extra_* and *Q_cell_* have been considered as well. It was carried out the fitting of the EIS diagrams and the results are given in Table 2.

This table shows that in the absence or in presence of cells, the heat treatment has no effect on the values of the solution resistance (~56.7 Ω cm^2^) as a result of the immersion time. The *Q_f_* values at 0 days are at the same order of magnitude (10^−5^ F cm^−2^) typical of passive films that slightly decrease as the oxide thickness is enhanced. Also, this parameter decreases by one order of magnitude (10^−6^ F cm^−2^) for Ti6Al4V, and by two (10^−7^ F cm^−2^) for the heat-treated samples immersed for 1 and 7 days; these results could be due to variations in the hydration of the TiO_2_, thickness and/or protein adsorption. This chemical adsorption modifies the non-ideal capacitance of the titanium oxide becoming more resistive through the immersion. In relation to the *R_f_* parameter, values of 10^7^ Ω cm^2^ are reached for the three samples at different immersion times, with these results being similar to those reported in the literature [56].

Addition of cells to the culture modifies the interface of the Ti alloys tested in this biological medium; thereby the non-ideal capacitance (*Q*_cell_) and resistance of the extracellular matrix (*R*_extra_) excreted by the cells on TiO_2_/adsorbed proteins can be analyzed. It is important to note that the *Q*_cell_ values are in the same order of magnitude (10^−5^ F cm^−2^) as those reported for the TiO_2_ (0 days). These findings can be explained by considering a weak adhesion and/or minor coverage of the osteoblast cells on the Ti6Al4V alloys (see below). Thus, it can be assumed that there is a poor interaction between the extracellular matrix excretion (mainly type I collagen) and the TiO_2_; likely due to its adsorption (via oxygen) through oxygen vacancies, as has been reported in the literature [16,26,31,53]. Regarding the *R*_extra_ values, a slight increase is seen at 1 day for Ti6Al4V as received, Ti6Al4V_800_ and Ti6Al4V_1050_ alloys, followed by its decrease after 7 days of immersion. This resistive contribution is related to the coverage of cells adhered to these surfaces, i.e., a greater cell adhesion would lead to an increase in this resistive term. Under this assumption, an enhanced coverage of the extracellular matrix and a higher proliferation of cells would be expected for Ti6Al4V as received, and to a minor extent for Ti6Al4V_800_ and Ti6Al4V_1050_ alloys (see below).

#### 2.4.3. Morphological Observation

**Figure 8:**
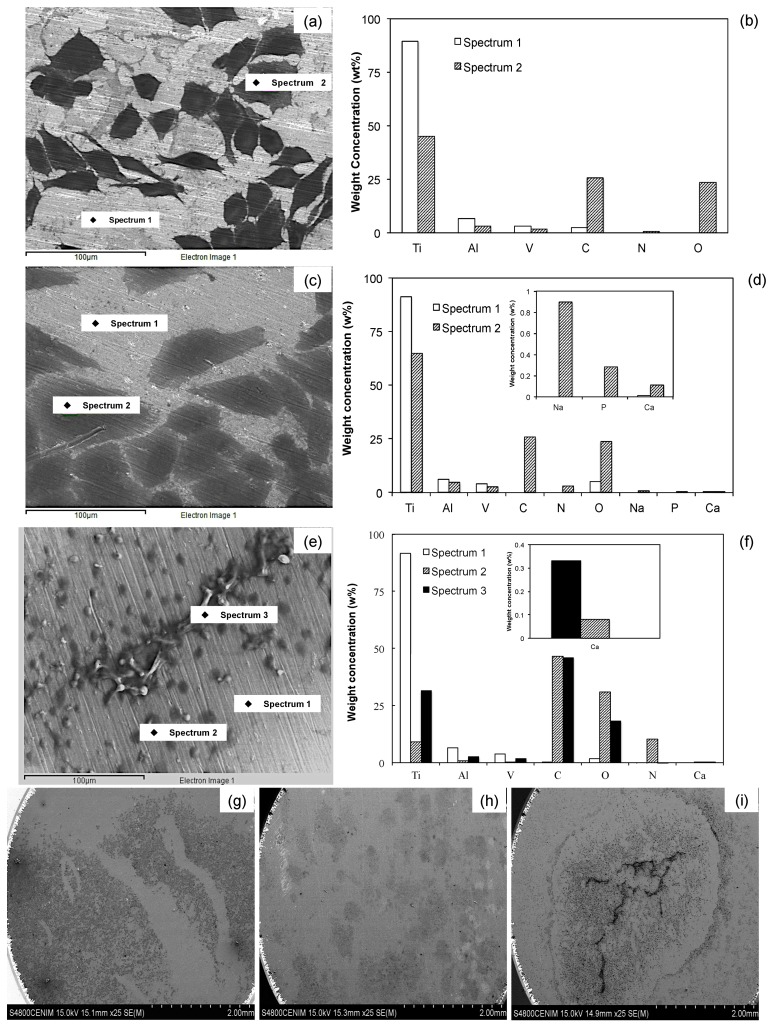
Overview of osteoblasts adhered and EDX analysis to Ti6Al4V as received (**a**,**b**,**g**), Ti6Al4V_800_ (**c**,**d**,**h**) and Ti6Al4V_1050_ (**e**,**f,i**) after 7 days of immersion in DMEM at 10% of FBS.

Figure 8 shows SEM images of the Ti surfaces after 7 days of immersion in the osteoblast culture medium. The three surfaces are partially covered by cells, which in the case of the Ti6Al4V as received and Ti6Al4V_800_ alloys are polygonal, elongated and fully spread (Figure 8a,c); whereas the Ti6Al4V_1050_ alloy (Figure 8e) presents round, non-spread cells, accumulated in some areas of the surface. This morphology could be due to the difficulties of cell adsorption on the oxide, which depends on the microstructure (lamellar microstructure) and distribution of alloyed elements (Al and V) after the heat treatment. The oxide film in the three alloys is partially covered by cells and extracellular matrix (Figure 8g–i) and there is an increase in osteoblast anchoring and proliferation in the following order: Ti6Al4V_1050_, Ti6Al4V_800_ and Ti6Al4V as received; consistent with the EIS analysis.

The element composition at the surface level (concentration in weight, %w) of different alloys was detected by EDX analysis after 7 days of immersion in the cell culture medium. In the analysis performed on cell-free areas of the three tested surfaces (Figure 8b,d,f), signals of Ti and O associated with the formation of TiO_2_ were detected; even though the O quantification was not evident for Ti6Al4V as received, because of the predominant Ti signal and low oxide thickness. Other metallic oxides (Al and V) may also form there, however their contribution in the oxide is minor because most of these signals come from the metallic substrate consistent with the XPS analysis. In the cell-covered areas, however, signals of C and O were identified, whose proportions were quite similar in Ti6Al4V as received and Ti6Al4V_800_ alloys; whereas in Ti6Al4V_1050_, the signal of C was larger than the signal of O. As the presence of these elements is related to cell adhesion, this process can be deemed to occur in a similar manner for Ti6Al4V as received and Ti6Al4V_800_; while for Ti6Al4V_1050_, the adhesion seems to take place differently, perhaps due to the synthesis of other carbon compounds from extracellular matrix and/or their orientation on the metallic surface [57,58]. To explain the reduced O signal, it can be suggested the O^−2^ adsorption from the chemical compounds could be retarded by the hydroxide or water species on the TiO_2_ surface being larger hydrated for Ti6Al4V_1050_. Besides, this adsorption process is affected by the formation of a less defective oxide on this alloy (less oxygen vacancies); conversely, for Ti6Al4V as received and Ti6Al4V_800_, the formation of other sub-oxides (TiO and Ti_2_O_3_) takes place to a major extent. Another difference between these alloys results from the presence of N, which is evident for Ti6Al4V_1050_, followed by Ti6Al4V_800_ and Ti6Al4V as received, and results from the presence of proteins, mainly type I collagen, but also from the presence of phosphorylated glycoproteins, osteocalcin and matrix Gla proteins. According to these results, it seems that the presence of organic compounds may vary during the process of cell adhesion due to the differences in the microstructure of the materials.

On the other hand, Ca (<0.33%w) and P (0.28%w) elements were detected on the surface of Ti6Al4V_800_ alloy, whereas on the Ti6Al4V_1050_ surface only Ca (0.11%w) was identified. Ca and P precipitation on these surfaces suggests that they are precursors of bone mineralization in in vivo assays and therefore, they play an important role in their interaction with metallic surfaces and consequent cell adhesion [58,59,60,61,62,63]. P and Ca were not detected on Ti6Al4V, likely due to a lower oxide hydration after 7 days of immersion, according to XPS results, see Figure 3. This assumption is consistent with studies on the biocompatibility and osteointegration of these materials, where oxide hydration had been reported to favor calcium phosphate precipitation [56,57,59,64].

The differences obtained during cell adhesion on Ti6Al4V as received, Ti6Al4V_800_ and Ti6Al4V_1050_ (Figure 8b,d,f) may be explained by suggesting that they are due to the interaction of proteins and/or cells with the oxide at surface level. The protein adsorption is the first stage prior to cell adhesion, whereas the quality of adhesion has an influence on the morphology, capacity for proliferation and cell differentiation [37,58,60,65]. So, taking into consideration the dominant phase in titanium alloys and morphology of cell adhesion, the adsorption process is considered to take place predominantly in domains where there is an α-phase (hcp); likely due to the presence of Al, which is widely known to be easily hydrated [47,54,66,67,68,69], enhancing the cell adhesion. Thus, depending on the microstructure, the proteins can orient on the oxide surface [42,58] and the morphology of the cell adhesion is different; e.g., the grains observed in Figure 1 for the Ti6Al4V as received and Ti6Al4V_800_ alloys encompass the same polygonal morphology of the cells. Conversely, for the Ti6Al4V_1050_ alloy, an enlarged cell adhesion is observed related to its lamellar features.

The proportion of sub-oxides that make up the passive oxide film after the sterilization process is an important point to highlight for the three tested alloys, because they are related to a less passive behavior of the Ti6Al4V as received. Conversely, a major passivation is obtained, e.g., for the Ti6Al4V_800_ and Ti6Al4V_1050_ alloys. Another important factor to consider is related to film hydration, which is enlarged for Ti6Al4V_1050_ (Figure 3g) compared to the other two alloys; this may be due to a reordering of alloy elements (Al and V) in the lamellar array obtained after heat treatment at 1050 °C [21]. The above facts may be related to Al enrichment in the α-phase [37,45,50] and to Ca adhesion to the surface of Ti6Al4V_1050_ alloy [40,57,62,63]. Furthermore, the presence of V in the oxide had been reported to inhibit the formation of calcium phosphate [69].

Figure 8g–i show that osteoblast adhesion and proliferation depend on the microstructure and the nature of the passive oxide on the substrate. These results indicate that cell adhesion takes place despite microstructural differences [70,71], depending on both their capacity for extra- and intracellular matrix excretion, and microstructural differences between Ti6Al4V as received, Ti6Al4V_800_ and Ti6Al4V_1050_ alloys.

## 3. Materials and Methods

### 3.1. Heat Treatments

Annealed Ti6Al4V alloy bars (Goodfellow Materials Ldt, Huntingdon, UK), 12.7 mm in diameter and 20 mm in length, were encapsulated in quartz under argon atmosphere to prevent their oxidation upon subjection to temperatures below (800 °C) and above (1050 °C) the β-phase transformation temperature (980 ± 20 °C) [33] for six hours, and later air-cooled (approximately 35 °C min^−1^). Afterwards, the heat-treated bars were cut to obtain 2 mm-thick discs.

### 3.2. Microstructure Revealing

The samples were prepared for microstructural observation following a standard metallographic technique: smoothing with 400-, 600-, 1200- and 1500-grit SiC paper sheet, polishing with 0.3 μm alumina to a mirror finish and chemical etch with Kroll’s reagent (HF + HNO_3_ and distilled water in 1:3:96 proportions) [34]. The microstructure was characterized using a Nikon EPIPHOT 300 optical microscope coupled to a Nikon FDX-35 camera (Nikon Instruments Europe B.V., Amsterdam, The Netherlands).

### 3.3. Electrochemical Cell

A glass electrochemical cell was specially designed for cell culture and consisted of a base, two Teflon plates, and a Ti6Al4V working electrode in between (with and without heat treatment). The electrochemical cell was screwed in the upper Teflon piece and adjusted with a silicone o-ring [64] and maintained at 37 °C in a water bath. A calomel-saturated electrode was utilized as a reference electrode, and a platinum wire as a counter electrode (Goodfellow Cambridge Ltd, Huntingdon, UK). Luer fittings were employed to control CO_2_ entry and exit (5%). The electrochemical cell and polished Ti6Al4V alloy discs, as received and heat-treated, and counter electrode, were sterilized in an autoclave for 30 min at 120 °C and 1.2 kg cm^−2^. The reference electrode was sterilized using UV-light for 10 min.

### 3.4. Cell Culture Medium

The cell culture medium (DMEM −10% FBS) was prepared as follows: 90 mL of DMEM (Dulbecco’s Modified Medium, Gibco® by life Technologies ™ Invitrogen GmbH, Darmstadt, Germany) were added 1 mL of L-Glutamine 200mM Gibco®, 1 mL of Penicillin-Streptomycin Gibco®, 1 mL of Sodium Pyruvate Gibco® and 10 mL of FSB (Fetal Bovine Serum, Sigma-Aldrich®, St. Louis, MO, USA).

### 3.5. *In Vitro* Assays

Saos-2 pre-osteoblast human osteosarcoma cells (from the cell bank of the Center for Biological Researches, CSIC, Madrid, Spain) were used for in vitro assays. After 24 h of immersion in culture medium, 10,000 cells were seeded on disks of Ti6Al4V as received, Ti6Al4V_800_ and Ti6Al4V_1050_ alloys, in order to cover the exposed metallic surface (A = 38.2 mm^2^). The assays lasted 7 days without and with electrical perturbation, at least in triplicate. In the first case, cells were only deposited and after the immersion, they were fixed for analysis using a Scanning Electron Microscope with microanalysis (SEM/EDX); whereas in the second case, open circuit potential (OCP) and Electrochemical Impedance Spectroscopy (EIS) measurements were made over time and the samples were later prepared and observed by SEM/EDX (not shown). It is worth mentioning that electrochemical measurements made at initial time (0 hours) were performed in the absence of cells, whereas the cell culture medium was renovated every 48 h to ensure an adequate contribution of nutrients to the cells and remove waste products from them. EIS characterization was carried out by applying a sinusoidal signal of 5 mV amplitude within frequency interval from 10^5^ Hz to 10^−2^ Hz with 10 points per decade, using a Gamry 600 Potentiostat-Galvanostat coupled to a PC (Gamry Instruments Inc, Warminster, PA, USA) for data acquisition and control.

### 3.6. Cell Fixation

To carry out morphological studies, cells seeded on metal discs immersed in 24-well plates for 7 days were fixed on the metal surface by adding 1 mL of 2% glutaraldehyde; which were kept at 4 °C for 24 h. Cells were then dehydrated by immersion in a series of ethanol solutions ranging from 35% to 100%. To dry the surface-adhered cells, a Trimethylsilane solution (TMS Sigma-Aldrich®) at 50% (0.5 mL of TMS in 0.5 mL of 100% ethanol) was added for 10 min. This solution was removed and 1 mL of TMS at 100% was added for another 10 min. Lastly, TMS was removed and left to air-dry during 30 min.

### 3.7. Surface Characterization

Surface analyses were carried out using the following devices: a Bruker AXS D8 Focus X-ray diffractometer (Bruker AXS GmbH, Karlsruhe, Germany) with Cu Kα radiation, fitted with an iron fluorescence filter, in the range 20°–90°, at a rate of 8° min^−1^ with a 0.02 passage. X-ray Mg/Al anode (CLAM 2) operating at 300W and at low pressures (< 10^−8^ Torr), whereas peaks were fitted with Gaussian-Lorentzian curves after subtracting the Sherly-type background using Leibol software (Homemade v. 2.0, CENIM, Madrid, Spain) and XPS (Fison Instruments, East Grinstead, UK). Hitachi S4800 Scanning Electron Microscope (Hitachi High-Technologies Corporation, Tokyo, Japan), operating at 15 kV and coupled to an EDX was used for the morphological observation.

## 4. Conclusions

This work has studied the properties of the oxide film formed on the surface of Ti6Al4V as received, Ti6Al4V_800_ and Ti6Al4V_1050_ alloys, immersed in osteoblast culture, using XRD, XPS and EIS techniques. The results show that depending on the temperature of heat treatment, the Ti6Al4V alloy can have two microstructures: globular and lamellar. The XRD analysis of these alloys allowed the identification of the α-phase as predominant in both microstructures. The XPS analysis determined that the passive oxide is mostly composed of TiO_2_, without discarding the presence of other sub-oxides (TiO, Ti_2_O_3_ and Al_2_O_3_). An important issue to emphasize is the decrease in sub-oxides, barely perceived in the case of Ti6Al4V_1050_. An increase in film thickness was seen in relation to the Ti6Al4V as received alloy, being double that of the heat-treated samples, which may be associated with the change in microstructure. The EIS technique allowed the observation of a stable passive behavior of the three materials immersed in cell culture for 7 days at 37 °C. As far as cell adhesion and proliferation are concerned, it was seen that cells are more dispersed on the surfaces of Ti6Al4V as received and Ti6Al4V_800_, whereas on Ti6Al4V_1050_ cells accumulated on the intermediate part of the surface. The above may be related to the nature of the substrate and the growth of passive oxide on it. The EDX analysis revealed Ca precipitated on Ti6Al4V_800_ and Ti6Al4V_1050_, however, the largest amount (0.3%w) was observed on the surface of Ti6Al4V_1050_ and in regions with the accumulation of cells. This is associated with hydration and the presence of Al in the outermost layer of the passive film. The microstructural changes in the Ti6Al4V alloy due to the heat treatments have an effect on the growth of the passive oxide, which leads to the variation of its chemical composition in relation to the presence of other sub-oxides, such as TiO, Ti_2_O_3_ and Al_2_O_3_. The above is directly related to cell adhesion and morphology as well as to cell proliferation. The presence of Ca and P on Ti6Al4V_800_ and TiAl4V_1050_ could indicate an important effect of the heat treatment on its biocompatibility. Therefore, these heat-treated surfaces could provide the Ti6Al4V alloy with an improvement in its performance as a biomaterial to use for the manufacture of orthopedic implants.

## Figures and Tables

**Table 1:** Oxide film thickness calculated using Strohmeier equation.

Sample	dTiO_2_ (nm)
Ti6Al4V as received	2.1
Ti6Al4V_800_	4.8
Ti6Al4V_1050_	5.0

**Table 2:** Parameter values obtained after the fitting of the EIS diagrams using the Boukamp program.

Sample	Time Days	Re (Ω cm^2^)	R_extra_ (Ω cm^2^)	Q_cell_ (Siemens s^n^) (cm^−2^)	*n*	R_f_ (Ω cm^2^)	Q_f_ (Siemens s^n^) (cm^−2^)	*n*	χ^2^
Ti6Al4V as received	0	57.74	---	---	---	1.91 × 10^7^	3.19 × 10^−5^	0.862	9.9 × 10^−3^
	1	46.13	381.2	2.82 × 10^−5^	0.887	1.78 × 10^7^	6.14 × 10^−6^	0.940	2.4 × 10^−3^
	7	50.78	146.7	2.69 × 10^−5^	0.900	1.76 × 10^7^	6.77 × 10^−6^	0.958	2.3 × 10^−3^
Ti6Al4V_800_	0	61.11	---	---	---	1.75 × 10^7^	2.25 × 10^−5^	0.882	5.6 × 10^−3^
	1	62.66	292.9	2.19 × 10^−5^	0.896	1.79 × 10^7^	2.06 × 10^−7^	0.907	2.4 × 10^−3^
	7	58.29	105	2.14 × 10^−5^	0.909	1.89 × 10^7^	2.83 × 10^−7^	0.919	2.2 × 10^−3^
Ti6Al4V_1050_	0	51.20	---	---	---	2.10 × 10^7^	2.20 × 10^−5^	0.913	6.6 × 10^−3^
	1	53.31	166.3	2.05 × 10^−5^	0.927	1.99 × 10^7^	2.89 × 10^−7^	0.939	2.7 × 10^−3^
	7	57.82	109.6	1.82 × 10^−5^	0.936	2.19 × 10^7^	2.81 × 10^−7^	0.957	1.9 × 10^−3^

## References

[1] Ratner B.D., Hoffman A.S., Schoen F.J., Lemons J.E. (2004). Biomaterials Science: An Introduction to Materials in Medicine.

[2] Scholz M.S., Blanchfield J.P., Bloom L.D., Coburn B.H., Elkington M., Fuller J.D., Gilbert M.E., Muflahi S.A., Pernice M.F., Rae S.I. (2011). The use of composite materials in modern orthopedic medicine and prosthetic device. Comps. Sci. Technol..

[3] Waite D.E. (1989). Overview and historical perspective of oral reconstructive surgery. Oral Surg. Oral Med. Oral Pathol..

[4] Lizuca T., Hallermann W., Seto I., Smolka W. (2006). A titanium arch bar for maxillomandibular fixation in oral and maxillofacial surgery. J. Oral Maxillofac. Surg..

[5] Castner D.G., Ratner B.D. (2002). Biomedical surface science: Foundations to frontiers. Surf. Sci..

[6] Sumita M. (1997). Present status and future trend of metallic materials used in orthopedics. Orthop. Surg..

[7] Liu X., Chu P.K., Ding C. (2004). Surface modification of titanium, titanium alloys and related materials for biomedical applications. Mater. Sci. Eng. R. Rep..

[8] Bhure R., Mahapatro A. (2010). Surface pattering using self-assembled monolayers (SAMs). Biomaterials.

[9] Williams D.F. (1981). Titanium and titanium alloys. Biocompatibility of Clinical Implant Materials.

[10] Goldberg J.R., Gilbert J.L. (2004). The electrochemical and mechanical behavior of passivated and TiN/AlN coated CoCrMo and Ti6Al4V alloys. Biomaterials.

[11] Ramires J., Guastaldi A.C. (2002). Study of Ti-6Al-4V biomaterial using electrochemistry and XPS techniques. Qui. Nova..

[12] Browne M., Gregson P.J. (2000). Effect of mechanical surface pretreatment on metal ion release. Biomaterials.

[13] Popa M.V., Demetrescu I., Vasilescu E., Drob P., Santana López A., Mirza-Rosca J., Vasilescu C., Ionita D. (2004). Corrosion susceptibility of implant materials Ti-5Al-4V and Ti-6Al-4Fe in artificial extra-cellular fluids. Electrochim. Acta..

[14] Contu F., Elsener B., Bohni H. (2002). Characterization of implant materials in fetal bovine serum and sodium-sulfate by electrochemical impedance spectroscopy-I-Mechanically polished samples. J. Biomed. Mater. Res..

[15] Chang E., Lee T.M. (2002). Effect of surface chemistry and characteristics of Ti6Al4V on the Ca and P adsorption and ion dissolution in Hanks ethylene diamine tetraacetic acid solution. Biomaterials.

[16] Frauchiger L., Taborelli M., Aronsson B.O., Descouts P. (1999). Ion adsorption on titanium surface exposed to a physiological solution. Appl. Surf. Sci..

[17] Jandt K.D. (2001). Atomic force microscopy of biomaterials surfaces and interfaces. Surf. Sci..

[18] Mathieu H.J. (2001). Bioengineered material-surfaces for medical applications. Surf. Interface Anal..

[19] Karimzadeh F., Heidarbeigy M., Saatchi A. (2008). Effect of heat treatment on corrosion behavior of Ti-6Al-4V alloy weldments. J. Mat. Process. Tech..

[20] Gil F.J., Fernández E., Arcas R., Planell J.A. (1994). Endurecimiento superficial mediante tratamiento térmicos y anodizado de la aleación Ti-6A1–4U para implantes quirúrgicos. Biomecánica.

[21] Geetha M., Kamachi Mudali U., Gogia A.K., Asokamani R., Raj B. (2004). Influence of microstructure and alloying elements on corrosion behavior of Ti-13Nb-13Zr alloy. Corros. Sci..

[22] Vinothkumar T.S., Miglani R., Lakshminarayananan L. (2007). Influence of deep dry cryogenic treatment on cutting efficiency and wear resistance of nickel–titanium rotary endodontic instruments. J. Endodontics.

[23] Wang X., Li Y., Lin J., Hodgson P.D., Wen C.E. (2008). Effect of heat-treatment atmosphere on the bond strength of apatite layer on Ti substrate. Dent. Mater..

[24] Feng K.C., Wu E.Y., Pan Y.N., Ou K.L. (2007). Effects of chemical and heat treatments on surface characteristics and biocompatibility of titanium-niobium alloys. Mat. Trans..

[25] Farhang P., Pupak A., Sirous A. (2012). Influence of mechanical and chemical surface treatments on the formation of bone-like structure in cpTi for endosseous dental implants. Appl. Surf. Sci..

[26] Lee B.H., Lee C., Kim D.G., Choi K., Lee K.H., Kim Y.H. (2008). Effect of surface structure on biomechanical properties and osseointegration. Mat. Sci. and Eng. C..

[27] Geetha M., Durgalaksshmi D., Asokamani R. (2010). Biomedical Implants: Corrosion and its Prevention-A Review. Recent Pat. Corros. Sci..

[28] Geetha M., Singh A.K., Asokamani R., Gogia A.K. (2009). Ti based biomaterials, the ultimate choice for orthopaedic implants–A review. Prog. Mater. Sci..

[29] Alipour R., Khani A., Mohammadi R., Rostami S. (2016). The effect of formation of titanium nitride thin film on surface characteristics of titanium by nitrogen ion implantation. J. Chem. Res..

[30] Cooper L.F., Masuda T., Whitson S.W., Yliheikkila P., Felton D.A. (1999). Formation of mineralizing osteoblast cultures on machined, titanium oxide grit–blasted, and plasma-sprayed titanium surfaces. Int. J. Oral Maxillofac. Implants.

[31] Mróz W., Bunder B., Syroka R., Niedzielski K., Golanski G., Slósarczyk A., Schwarz D., Douglas T.E. (2015). In vivo implantation of porous titanium alloy implants coated with magnesium-doped octacalcium phosphate and hydroxyapatite thin films using pulsed laser deposition. J. Biomed. Mater. Res. B.

[32] Sing S.L., Yeong W.Y., Wiria F.E., Tay B.Y. (2016). Characterization of titanium lattice structures fabricated by selective laser melting using an adapted compressive test method. Exp. Mech..

[33] Vydehi Arun J. (2006). Titanium Alloys: An Atlas of Structures and Fracture Features, Physical Metallurgy of Titanium Alloys.

[34] Donachie M.J. (2000). Titanium a Technical Guide. Understanding the Metallurgy of Titanium.

[35] Rack H.J., Qazi J.I. (2006). Titanium alloys for biomedical applications. Mat. Sci. Eng. C.

[36] Hanawa T., Asami K., Asaoka K. (1998). Repassivation of titanium and surface oxide film regenerated in simulated bioliquid. J. Biomed. Mater. Res. A.

[37] Da Fonseca C., Boudin S., da Cunha-Belo M.J. (1994). Characterization of titanium passivation films by in situ ac impedance measurements and XPS analysis. J. Electroanal. Chem..

[38] Vrancken B., Thijs L., Kruth J.P., van Humbeeck J. (2012). Heat treatment of Ti6Al4V produced by Selective Laser Melting: Microstructure and mechanical properties. J. Alloys Comp..

[39] Sallica-Leva E., Jardini A.L., Fogagnolo J.B. (2013). Microstructure and mechanical behavior of porous Ti–6Al–4V parts obtained by selective laser melting. J. Mech. Behav. Biomed..

[40] Li S.J., Yang R., Niinomi M., Hao Y.L., Cui Y.Y. (2004). Formation and growth of calcium phosphate on the surface of oxided Ti-29Nb-13Ta-4.6Zr alloy. Biomaterials.

[41] Malinov S., Guo Z., Sha W., Wilson A. (2001). Differential scanning calorimetry study and computer modelling of β ⇒ α phase transformation in Ti-6Al-4VAlloy. Met. Mat. Trans. A.

[42] Malinov S., Sha W., Guo Z., Tang C.C., Long A.E. (2002). Synchrotron X-ray diffraction study of the phase transformations in titanium alloys. Mater. Charact..

[43] Elmer J.W., Palmer T.A., Babu S.S., Specht E.D. (2005). In situ observations of lattice expansion and transformation rates of α and β phases in Ti–6Al–4V. Mat. Sci. Eng. A.

[44] Feliu S., Barranco V. (2003). XPS study of the surface chemistry of conventional hot-dip galvanised pure Zn, galvanneal and Zn–Al alloy coatings on steel. Acta Mater..

[45] Variola F., Yi J.H., Richert L., Wuest J.D., Rosei F., Nanci A. (2008). Tailoring the surface properties of Ti6Al4V by controlled chemical oxidation. Biomaterials.

[46] Tanaka Y., Nakai M., Akahori T., Niinomi M., Tsutsumi Y., Doi H., Hanawa T. (2008). Characterization of air-formed surface oxide film on Ti–29Nb–13Ta–4.6Zr alloy surface using XPS and AES. Corros. Sci..

[47] Bunker B.C., Nelson G.C., Zavadil K.R., Barbour J.C., Wall F.D., Sullivan J.P. (2002). Hydration of passive oxide films on aluminum. J. Phys. Chem. B.

[48] Armstrong N.R. (1977). Auger and X-Ray Photoelectron spectroscopic and electrochemical characterization of titanium thin film electrodes. Surf. Sci..

[49] Strohmeier B.R. (1990). An ESCA method for determining the oxide thickness on aluminium-alloys. Surf. Interface Anal..

[50] Milosev I., Metikos-Hukovic M., Strehblow H.H. (2000). Passive film on orthopaedic TiAlV alloy formed in physiological solution investigated by X-ray photoelectron spectroscopy. Biomaterials.

[51] Hiromoto S., Hanawa T. (2004). pH near cells on stainless steel and titanium. Electrochem. Solid State Lett..

[52] Hiromoto S. (2008). Corrosion of metallic biomaterials in cell culture environments. Electrochem. Soc. Interface.

[53] De Assis S.L., Wolynec S., Costa I. (2006). Corrosion characterization of titanium alloys by electrochemical techniques. Electrochim. Acta..

[54] Souto M.R., Laz M.M., Reis R.L. (2003). Degradation characteristics of hydroxyapatite coatings on orthopaedic TiAlV in simulated physiological media investigated by electrochemical impedance spectroscopy. Biomaterials.

[55] Lavos-Valereto I.C., Wolynec S., Ramires I., Guastaldi A.C., Costa I. (2004). Electrochemical impedance spectroscopy characterization of passive film formed on implant Ti6Al7Nb alloy in Hank’s solution. J. Mater. Sci.: Mater. Med..

[56] Chikarakara E., Fitzpatrick P., Moore E., Levingstone T., Grehan L., Higginbotham C., Vázquez M., Bagga K., Naher S., Brabazon D. (2016). In vitro fibroblast and pre-osteoblastic cellular responses on laser surface modified Ti–6Al–4V. Appl. Surf. Sci..

[57] Kubies D., Himmlová L., Riedel T., Chánová E., Balík K., Douděrová M., Bártová J., Pešáková V. (2011). The Interaction of Osteoblasts with Bone-Implant Materials: 1. The effects of physicochemical surface properties of implant materials. Physiol. Res..

[58] Anselme K. (2000). Osteoblast adhesion on biomaterials. Biomaterials.

[59] Castellani C., Lindtner R.A., Hausbrandt P., Tschegg E., Stanzl-Tschegg S.E., Zanoni G., Beck S., Weinberg A.M. (2011). Bone–implant interface strength and osseointegration: Biodegradable magnesium alloy versus standard titanium control. Acta Biomater..

[60] Mustafa K., Pan J., Wroblewsli J., Leygraf C., Arvidson K. (2002). Electrochemical impedance spectroscopy and X-ray photoelectron spectroscopy analysis of titanium surfaces cultured with osteoblast-like cells derived from human mandibular bone. J. Biomed. Mater. Res..

[61] Boyan B.D., Hummert T.W., Dean D.D., Schwartz Z. (1996). Role of material surfaces in regulating bone and cartilage cell response. Biomaterials.

[62] Keller J.C., Stanford C.M., Wightman J.P., Draughn R.A., Zaharias R. (1994). Characterizations of titanium implant surface III. J. Biomed. Mater. Res..

[63] Chesmel K.D., Clark C.C., Brighton C.T., Black J. (1995). Cellular responses to chemical and morphologic aspects of biomaterial surfaces. II. The biosynthetic and migratory response of bone cell populations. J. Biomed. Mater. Res..

[64] Lausma J., Kasemo B. (1990). Surface spectroscopic characterization on titanium implant materials. Appl. Surf. Sci..

[65] García-Alonso M.C., Saldaña L., Alonso C., Barranco V., Muñoz-Morris M.A., Escudero M.L. (2009). In situ cell culture monitoring on a Ti–6Al–4V surface by electrochemical techniques. Acta Biomater..

[66] Göpel W., Anderson J.A., Frenkel D., Jeaning M., Philips K., Schäfer J.A., Rocker G. (1984). Surface defects of TiO_2_ (1 1 0): a combined XPS, XAES and ELS study. Surf. Sci..

[67] Acevedo-Peña P., Vázquez-Arenas J., Cabrera-Sierra R., Lartundo-Rojas L., González I. (2013). Ti anodization in alkaline electrolyte: The relationship between transport of defects, film hydration and composition. J. Electrochem. Soc..

[68] Acevedo-Peña P., Vázquez-Arenas J., Cabrera-Sierra R., Lartundo-Rojas L., González I. (2013). Hydration and structural transformations during titanium anodization under alkaline conditions. ECS Trans..

[69] Hanawa T., Hiromoto S., Asami K., Okuno O., Asaoka K. (2002). Surface oxide films on titanium alloys regenerated in Hank’s solution. Mater. Trans..

[70] Calderón-Moreno J.M., Vasilescu C., Drob S.I., Ivanescu S., Osiceanu P., Drob P., Popa M., Preda S., Vasilescu E. (2014). Microstructural and mechanical properties, surface and electrochemical characterization of a new Ti-Zr-Nb alloy for implant applications. J. Alloys Compd..

[71] Niinomi M. (2003). Fatigue performance and cytotoxicity of low rigidity titanium alloy Ti-29Nb-13Ta-4.6Zr. Biomaterials.

